# Cholera Toxin Subunit B for Sensitive and Rapid Determination of Exosomes by Gel Filtration

**DOI:** 10.3390/membranes10080172

**Published:** 2020-07-31

**Authors:** Karolina Sapoń, Dominika Maziarz, Teresa Janas, Aleksander F. Sikorski, Tadeusz Janas

**Affiliations:** 1 Institute of Biology, University of Opole, Kominka 6, 45-032 Opole, Poland; karolina.sapon@uni.opole.pl (K.S.); dominika.maziarz@o2.pl (D.M.); teresa.janas@uni.opole.pl (T.J.); 2 Research and Development Center, Regional Specialist Hospital, 51-124 Wroclaw, Poland; aleksander.sikorski@uwr.edu.pl; 3 Department of Cytobiochemistry, Faculty of Biotechnology, University of Wrocław, 50-383 Wrocław, Poland

**Keywords:** cholera toxin subunit B, cholesterol, exosomes, gel chromatography, GM1 ganglioside, liposomes

## Abstract

We developed a sensitive fluorescence-based assay for determination of exosome concentration. In our assay, Cholera toxin subunit B (CTB) conjugated to a fluorescence probe and a gel filtration technique (size-exclusion chromatography) are used. Exosomal membranes are particularly enriched in raft-forming lipids (cholesterol, sphingolipids, and saturated phospholipids) and in GM1 ganglioside. CTB binds specifically and with high affinity to exosomal GM1 ganglioside residing in rafts only, and it has long been the probe of choice for membrane rafts. The CTB-gel filtration assay allows for detection of as little as 3 × 10^8^ isolated exosomes/mL in a standard fluorometer, which has a sensitivity comparable to other methods using advanced instrumentation. The linear quantitation range for CTB-gel filtration assay extends over one order of magnitude in exosome concentration. Using 80 nM fluorescence-labeled CTB, we quantitated 3 × 10^8^ to 6 × 10^9^ exosomes/mL. The assay ranges exhibited linear fluorescence increases versus exosome concentration (r^2^ = 0.987). The assay was verified for exosomal liposomes. The assay is easy to use, rapid, and does not require any expensive or sophisticated instrumentation.

## 1. Introduction

Extracellular vesicles (EVs) originate from various types of cells. In mammals, they are involved in disordered physiological processes including cardiovascular diseases, cancer, regeneration, and immune responses. Exosomes belong to the smallest group of EVs (sEVs), they are formed within multivesicular bodies (MVBs) as intraluminal vesicles. Exosomes (50–150 nm in diameter) are released from cells when MVBs fuse, at the cytoplasmic site, with the plasma membrane. EVs larger than exosomes (microvesicles, also known as ectosomes or shedding vesicles), 100–1000 nm in diameter, are released from cells through blebbing (budding out) and fission of the plasma membrane. The largest EVs (apoptotic bodies), 0.5–2 μm in diameter, are derived from cells during apoptosis. Exosomes contain nucleic acids (including miRNAs), proteins (including glycoproteins), and lipids (including glycosphingolipids) [1,2]. Exosomal membranes are particularly enriched in raft-forming lipids (cholesterol, sphingolipids, and saturated phospholipids) and in GM1 ganglioside [3,4]. Exosomes have important role in the pathogenesis of neurodegenerative disease, as well as in the function of normal nervous system [5]. In cancer, exosomes seem to influence both microenvironments at distant sites, thus facilitating successful metastasis and the tumor microenvironment surrounding closely the primary tumor [6].

Cholera toxin subunit B (CTB) is the nontoxic portion of cholera toxin secreted by Gram-negative bacteria *Vibrio cholerae*. [7]. CTB is composed of five CTB monomers and forms a ring-like structure. GM1 is the receptor for CTB, and its recognition regulates the cellular uptake of CTB. CTB specifically interacts with GM1 residing in membrane rafts; therefore, it is used as a lipid probe for membrane rafts. Each monomer of CTB interacts with one pentasaccharide moiety of GM1; however, adjacent CTB molecules are also in contact with this pentasaccharide moiety of GM1 [8].

The value of quantifying exosomes is indispensable, since it is necessary for quantitative measurements, e.g., dissociation constant of exosome-ligand complex, and also for comparison of results from different preparations within the same report or different reports. Quantification of sEVs has been achieved by cryo-electron microscopy, by a platform combining surface plasmon resonance with high-resolution flow cytometry, atomic force microscopy, by resistive pulse sensing, and by methods based on nanoparticle tracking analysis [9]. However, because of the lack of cost-effective analytical protocol quantification of sEVs, it still remains a challenge [10]. These assays require access to expensive instrumentation and advanced technical skills, and also involve time-consuming procedures for sample preparations. While the combination of fluorescence detection and gel filtration for determination of EVs has been described to some extent before, prior studies have been limited to non-specific membrane dyes [9] that have limited applicability for analyzing exosomes in complex body fluids, or limited to a combination of gel filtration and antibody-labeling with a sophisticated microfluidic resistive pulse sensing technique [11].

In this study, we developed and validated gel chromatography for analysis of exosomes using an exosome-specific label (CTB) in complex media. Columns filled with S-1000 Sephacryl were evaluated to obtain a fast separation of exosomes and also a high recovery. We applied separation of exosomes from other endogenous components, e.g., lipoproteins and amino acids, by gel filtration. We report a development of a cost effective protocol for the quantification of exosomes. In order to achieve high assay sensitivity, fluorescence detection was deployed for monitoring the eluent for the presence of fluorescence-labeled CTB attached to exosomes.

## 2. Materials and Methods

### 2.1. Chemicals and Materials

Lipids: cholesterol (CHOL); *N*-stearoyl-D-erythro-sphingosylphosphorylcholine (Stearoyl Sphingomyelin, SM); 1,2-dioleoyl-sn-glycero-3-phosphocholine (DOPC); 1,2-dipalmitoyl-sn-glycero-3-phosphocholine (DPPC); 1,2-dioleoyl-sn-glycero-3-phospho-L-serine (DOPS); 1,2-dipalmitoyl-sn-glycero-3-phospho-L-serine (DPPS); 1,2-dioleoyl-sn-glycero-3-phosphoethanolamine (DOPE); 1,2-dipalmitoyl-sn-glycero-3-phosphoethanolamine (DPPE); and bovine brain ganglioside GM1 were purchased from Avanti Polar Lipids (Alabaster, AL, USA). Amplex Red Cholesterol Assay Kit and fluorescent probe Cholera Toxin subunit B (recombinant) Alexa Fluor 555 conjugate (CTB555) were purchased from ThermoFisher Scientific (Waltham, MA, USA). Fetal Bovine Serum (FBS) was purchased from Sigma-Aldrich (St. Louis, MO, USA). Sephacryl S-1000 superfine was purchased from Sigma-Aldrich (St. Louis, MO, USA). Millipore Ultrafree-MC Centrifugal Filter Devices with microporous membranes 100 nm pore size were purchased from VWR (Radnor, PA, USA).

### 2.2. Isolation of Exosomes

Differential ultracentrifugation, size-exclusion chromatography, and ultrafiltration have been used in consecutive three steps for exosome purification from FBS, as described [12]. Differential ultracentrifugation: 20 min centrifugation of FBS (total volume 30 mL) at 2000× *g*, then 18 h centrifugation of the supernatant at 126,000× *g* (Type 45 Ti rotor, Beckman Coulter, Pasadena, CA, USA), washing the pellet with PBS and resuspending with PBS, re-pelleting it by 70 min ultracentrifugation at 126,000× *g*, finally resuspending in 200 μL PBS. Size-exclusion chromatography: 1.1 mL Sephacryl S-1000 gel filtration column (diameter 4.7 mm, height 70 mm). Ultrafiltration: 100-nm pore size ultrafiltration (final exosome purification) using Millipore Ultrafree-MC Centrifugal Filter Device (centrifugation at 12,000× *g*, 1 min). The size distribution and homogeneity of exosomes have been determined by using Zetasizer Nano ZS (Malvern Instruments Ltd., Malvern, Worcestershire, UK), the diameter distribution was 50–110 nm [12]. Exosomes from neuroblastoma IMR-32 cell culture supernatants were purified as described [12].

### 2.3. Preparation of Exosomal Liposomes

Liposomes were prepared as described [13]. Briefly, lipid solutions in chloroform/methanol (2/1) were mixed, solvent was evaporated under a stream of nitrogen, and then the dried lipid film was further desiccated under vacuum for 2 h at room temperature. The lipids (CHOL 43.5 mol%, SM 18 mol%, DOPC 8 mol%, DPPC 8 mol%, DOPS 7 mol%, DPPS 7 mol%, DOPE 4 mol%, DPPE 4 mol%, and GM1 0.5 mol%) were first resuspended in PBS, and then multilamellar liposomes were formed by gentle vortex. Liposome suspension was subjected to seven freeze–thaw cycles, and large unilamellar vesicles (LUVs) were prepared by extrusion using the Avanti MiniExtruder with a filter pore diameter of 100 nm. The lipid composition of these LUVs resembles the lipid composition of exosomes [3,14] in relation to the level of cholesterol, sphingomyelin, unsaturated and saturated phospholipid, negative charge of phospholipids, and the presence of gangliosides; therefore, they are designated as “exosomal liposomes”. The size distribution and homogeneity of liposomes have been determined by using Zetasizer Nano ZS (Malvern Instruments Ltd., Malvern, Worcestershire, UK), the diameter distribution was 60–160 nm.

### 2.4. Gel Filtration Assay

**Figure 1:**
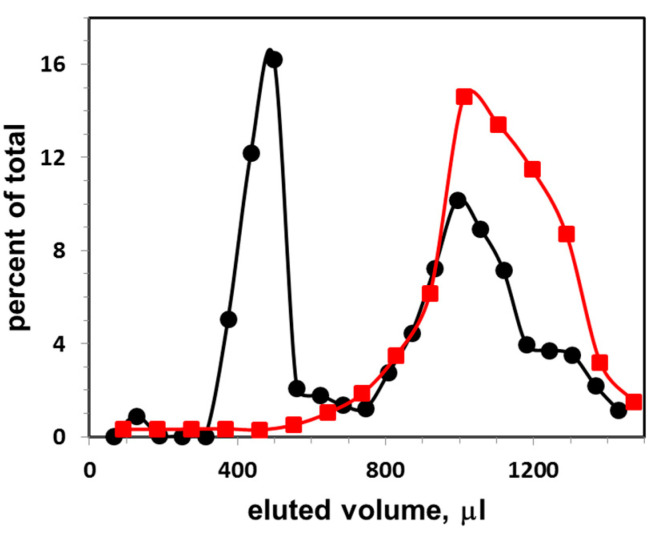
Gel filtration elution profile of exosomes (at concentration 6 × 10^9^ particles per mL) isolated from FBS, and next incubated with CTB555: vertical axis, percent of total fluorescence; horizontal axis, eluted volume. The excitation and emission wavelengths of the fluorescence were λ_ex_/λ_em_ = 545/565 nm. Black circles, distribution of CTB555 between exosomes and buffer; red squares, distribution of CTB555 when eluted without exosomes.

In order to separate CTB-labeled exosomes (or liposomes) from free (unbound) CTB, gel filtration assay was used. Gel filtration assay was performed as described [15]. Briefly, 40 μL of exosomes (or liposomes) were incubated with CTB555 (80 nM) for 1 h at 37 °C, then applied to 1 mL S-1000 column and eluted with PBS. CTB555 specifically binds to GM1 ganglioside located within the raft-like region of exosome membrane [13,14]. The optimal CTB concentration for exosome detection depends on the sensitivity of the spectrofluoromer and resolution of the gel filtration column used during the procedure. In our case, a tenfold decrease of CTB results in too low fluorescence intensity of the samples, and a tenfold increase in CTB concentration results in a too large second peak (non-bound CBT555, see Figure 1) overlapping the first (exosomal) peak. The eluent was collected into tubes (ca 2 drops per one tube), and the samples were analyzed for fluorescence using Cary Eclipse spectrofluorometer with cuvette holder maintained at 37 °C. Exosomes (or liposomes) without fluorescence probes were used to correct for light scattering.

### 2.5. Exosome Quantification

We measured the cholesterol concentration of the exosome suspension using an Amplex Red cholesterol assay kit, according to the manufacturer’s instruction. We determined the concentration of exosomes from this cholesterol level. For calculations, we assumed that cholesterol constituted ca. 43 mol% of total lipids [14], and membrane proteins cover ca. 30% of the membrane surface [16,17,18]. In the case of our exosomes, there are ca. 24,000 cholesterol molecules per one 80-nm diameter exosome, as our calculations revealed. Typical calculations revealed that one 80-nm (in diameter) exosome has a total (external + internal) surface of 35,500 nm^2^, and cholesterol covers 20% of the exosomal surface.

### 2.6. Liposome Quantification

Liposome concentration was calculated assuming that the area per one lipid molecule in lipid bilayer is 0.3 nm^2^, 0.5 nm^2^, 0.5 nm^2^, and 0.61 nm^2^ for cholesterol, sphingolipids, saturated phospholipids, and unsaturated phospholipids, respectively [19,20]. In our exosomal liposomes, there are ca. 140,000 lipid molecules (including 61,000 cholesterol molecules) per one 100-nm diameter liposome, according to our calculations. Typical calculations revealed that one ca. 100-nm (in diameter) liposome has a total (external + internal) surface of 60,000 nm^2^, and cholesterol covers 29% of the liposomal surface.

## 3. Results

### 3.1. Gel Filtration of Exosomes

Figure 1 presents the elution profile of exosomes obtained from FBS. The exosomes were first incubated with CTB555, then they were applied on the gel filtration column and eluted with PBS buffer (black circles). Percent of total fluorescence intensity as a function of eluted volume is shown. The first peak represents the liposome-CTB555 complexes, while the second peak represents the free CTB555. The sum of fluorescence intensities of three points (with highest fluorescence intensities) of the exosome peak was calculated, and this sum was taken as a one-point-data for the exosome calibration curve. As a control, an elution profile of CTB555 without exosomes is presented (red squares): the elution peak co-localizes with the second elution peak of the mixture of exosome-CTB555 complexes and non-bound CTB.

### 3.2. Calibration Curve for Exosome Quantification

**Figure 2:**
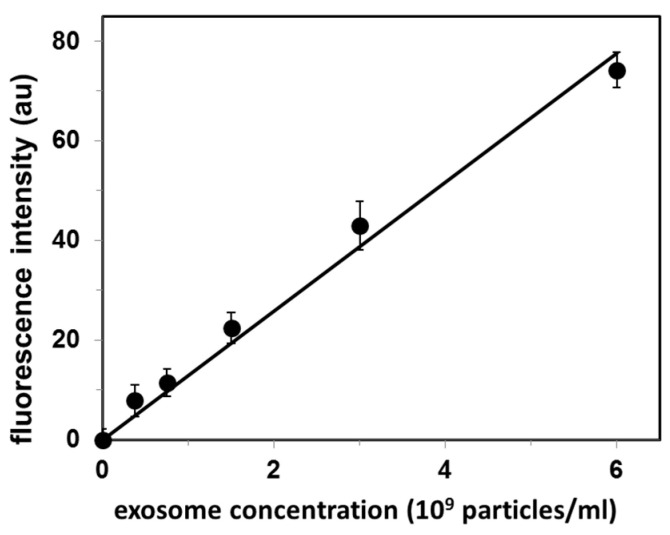
Calibration curve for quantification of exosomes isolated from fetal bovine serum (FBS). Values are mean ± SE of 3 independent experiments. Fluorescence intensity is in arbitrary units (au).

Exosome concentration (as exosome particles/mL) was determined on the basis of cholesterol level measured for the exosome suspension and the cholesterol standard curve obtained by using the Amplex Red cholesterol assay kit. This sample was used as a stock exosome solution, and sequentially diluted to prepare a set of standard exosome solutions. Analysis of these standard solutions by our CTB555-gel filtration method produced a linear calibration curve (r^2^ = 0.987), as shown in Figure 2.

The original exosome concentration in FBS determined from the standard curve (Figure 2) was 4.0 × 10^8^ exosomes/mL. The upper limit of detection reported in Figure 2 (6 × 10^9^ exosomes/mL) results from our experimental procedure for exosome isolation/concentration: this is the maximal exosomal concentration that we can obtain.

We have also performed another control: we used exosomes obtained from different source, i.e., from neuroblastoma IMR-32 cell culture. In order to determine the unknown concentration of exosomes, the sample was first incubated with fluorescent-labeled CTB, next passed through S-1000 gel filtration column, and finally the fluorescence of the exosomal peak was measured. This fluorescence intensity value was used to determine the exosome concentration from the calibration curve, and the apparent concentration of (2.1 ± 0.2) × 10^9^ and (1.4 ± 0.1) × 10^9^ exosomes/mL (mean ± SE for N = 3 experiments) for two different exosome preparations. As confirmed by cholesterol level measurements [(2.2 ± 0.3) × 10^9^ and (1.3 ± 0.3) × 10^9^ exosomes/mL (mean ± SE for N = 3 experiments), respectively], the exosome concentration from the calibration curve was a correct (within the experimental error) determination of these exosome concentrations.

### 3.3. Gel Filtration of Exosomal Liposomes

**Figure 3:**
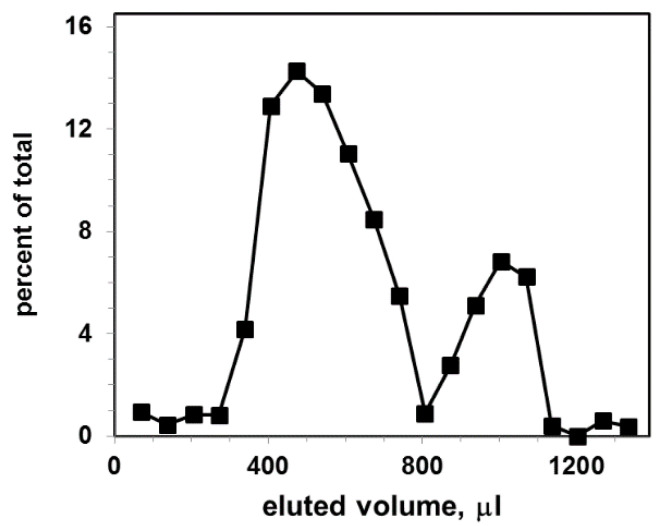
Gel filtration elution profile for exosomal liposomes (concentration 9 × 10^9^ particles per mL) after incubation with CTB555: vertical axis, percent of total fluorescence; horizontal axis, eluted volume. The excitation and emission wavelengths of the fluorescence were λ_ex_/λ_em_ = 545/565 nm.

Figure 3 shows the elution profile of labeled with CTB555 exosomal liposome dispersion.

The Sephacryl S-1000 column separates CTB555 bound to liposomes (first peak) from non-bound CTB555 (second peak). As a result, two distinct elution peaks, shifted relative to each other, were obtained: the first represents liposome-CTB complexes, the second represents non-bound CTB555. The size distribution of liposomes is larger in comparison with exosomes; therefore, the liposomal gel filtration first peak is broader than the exosomal first peak.

### 3.4. Calibration Curve for Exosomal Liposome Quantification

**Figure 4:**
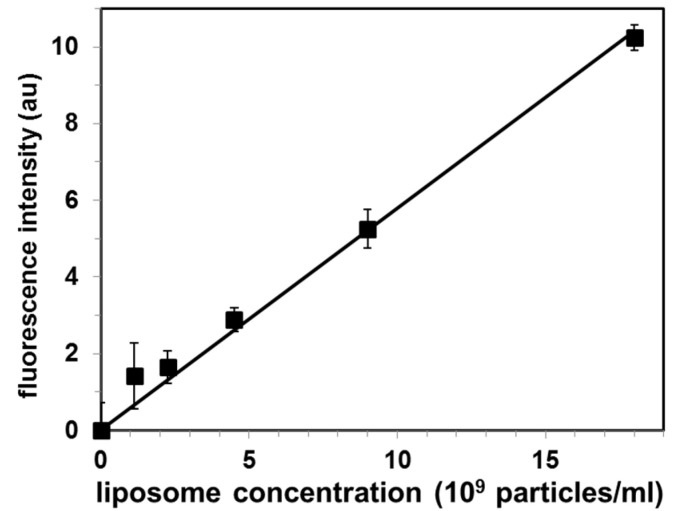
Calibration curve for quantification of exosomal liposomes by the proposed Cholera toxin subunit B (CTB)-gel filtration method. Values are mean ± SE of 3 independent experiments. Fluorescence intensity is in arbitrary units (au).

In order to determine the exosomal liposome concentration (as liposome particles/mL), we used the cholesterol level of the liposome suspension and the cholesterol standard curve, by using the Amplex Red cholesterol assay kit. This sample was used as a stock liposome solution, and sequentially diluted to prepare a set of standard liposome solutions. The total fluorescence intensity of the liposomal peak (calculated as the sum of the three highest fluorescence intensities within the liposomal peak in Figure 3) as a function of the liposome concentration is shown in Figure 4.

Each point represents independent gel filtration assays (mean ± SE for N = 3 experiments) Analysis of these standard solutions by our CTB555-gel filtration method produced a linear calibration (r^2^ = 0.988) curve, as shown in Figure 4.

## 4. Discussion

In this study, we developed a sensitive and rapid method for the determination of exosomes which does not require sophisticated or expensive devices. We combined the high affinity and sensitivity of Cholera toxin subunit B (CTB) for exosomal GM1 ganglioside and Sephacryl S-1000 gel filtration, first to label exosomes with CTB conjugated to a fluorescence probe (CTB555), and then to separate these labeled exosomes from non-bound CTB555. CTB monomers spontaneously associate in water solution into pentamers and a role of protonation of histidine residues is suggested in this process [21]. The assay ranges exhibited linear fluorescence increases versus exosome concentration (r^2^ = 0.987). The obtained range of exosome concentration (3 × 10^8^–6 × 10^9^ exosomes/mL) in the calibration curve using our CTB-gel filtration method, is similar to the range obtained for small extracellular vesicles (sEVs) labeled with a non-specific fluorescence probe CM-Dil [9], and it is also similar to another reported method [11] utilizing a combination of gel filtration of EVs labeled with anti-CD235a antibody, specific to glycophorin A, with a sophisticated microfluidic resistive pulse sensing technique. The physiological range of exosome concentrations in serum/plasma is 10^10^–10^11^ exosomes/mL [22], and in the case of cell culture conditioned media, up to 10^10^ exosomes/mL, depending on the time duration of the cell culture [10,23].

We verified the CTB-gel filtration method, using exosomal liposomes as a model for exosomes. These liposomes have the following properties: first, they are composed of major lipids present within the exosome membrane; second, they contain similar concentration of these major lipids in comparison to exosome membrane; and third, they have a similar diameter in comparison with exosomes. We noticed an interesting phenomenon: the fluorescence intensity of exosomes is 22-fold higher in comparison with exosomal liposomes at the same concentrations. A similar phenomenon was observed in the case of the complex formed by CTB and GM1 embedded in solid-supported bilayer lipid membranes made of sphingomyelin and cholesterol [24]. The value of the dissociation constant deviated one order of magnitude from that measured for in vivo cells, as in our experiments with liposomes and exosomes. CTB binds with high affinity (K_D_ < 1 nM) to GM1 ganglioside [8]. However, recent work suggests that CTB may not bind exclusively to GM1 [25]. It was demonstrated that fucosylated glycoproteins mediated a large portion of CTB binding to human colonic epithelial cell lines, and it was the glycan component, not the protein itself, that interacted with CTB. Therefore, we suggest that, in the case of our liposomal exosomes, CTB binds only to GM1; however, in the case of natural membranes (plasma membrane and exosomal membrane), CTB binds to both GM1 and fucosylated glycoproteins, hence the affinity of CTB to natural membranes is higher in comparison with model lipid membranes (lipid bilayers and liposomes). Fucosylated N-glycans were found on glycoproteins in tumor-derived EVs [26,27,28].

This determination of exosomes by gel filtration is especially easy to perform for labs using gel chromatography for exosome purification. Once the calibration curve is measured and plotted, this calibration curve can be used for sensitive and rapid determination of exosomes for several months, using Cholera toxin subunit B, assuming that the same gel chromatography protocol is used and that the same spectrofluorometer with setting is used. The generation of a calibration curve requires the exosome concentration to be determined by another method, which can be, for instance, cholesterol content (as in our report), protein content [10], or dynamic light scattering using a particle counter [22]. Alternatively, the exosomal liposome suspension can be used to generate the liposome calibration curve. Since both curves are linear with intercept (0,0), and the slope of exosomal line is 22-fold bigger than the slope of the liposomal line, the calibration curve for exosomal liposomes can be used for the determination of the exosome concentration: the apparent exosome concentration derived from the liposomal calibration curve should be divided by 22, in order to obtain the concentration of exosomes.

The calibration curve, generated for FBS exosomes turned out to be correct in the case of exosomes purified from neuroblastoma cell culture. This phenomenon can be explained by a similar percentage of GM1 lipids in exosomal membrane lipids, and also possibly from a similar level of fucosylated glycoproteins. It seems that the concentration of exosomes released from some other cell cultures can be determined from the calibration curve of FBS exosomes. However, a control test should be performed first, as in our case of neuroblastoma exosomes.

## 5. Conclusions

In summary, we developed a CTB-gel filtration assay which allows the detection of as little as 3 × 10^8^ exosomes/mL in a standard fluorometer. The linear quantitation range for our assay extends over one order of magnitude in exosome concentration. Using 80 nM CTB555, we quantitated exosomes within the range from 3 × 10^8^ to 6 × 10^9^ exosomes/mL. The assay is easy to use, rapid, and does not require any expensive or sophisticated instrumentation.
